# Editorial: Methodological and technical issues of tele-neuropsychology: remote cognitive assessment and intervention across the life span

**DOI:** 10.3389/fpsyg.2025.1745809

**Published:** 2025-12-08

**Authors:** Laura Veronelli, Scott A. Sperling, Sara Mondini

**Affiliations:** 1 Department of Psychology, University of Milano-Bicocca, Milan, Italy; 2 Department of Neurorehabilitation Sciences, Casa di Cura IGEA, Milan, Italy; 3 Center for Neurological Restoration, Neurological Institute, Cleveland Clinic, Cleveland, OH, United States; 4 Department of Philosophy, Sociology, Education and Applied Psychology (FISPPA), University of Padua, Padua, Italy; 5 IRCCS San Camillo Hospital, Venice, Italy

**Keywords:** tele-neuropsychology, remote cognitive assessment, remote cognitive interventions, tele-medicine, tele-health, digital literacy

Tele-neuropsychology (t-NP) is defined “as the application of audiovisual technologies to enable remote clinical encounters with patients to conduct neuropsychological assessments” (Bilder et al., 2020, p. 648; through the Inter Organizational Practice Committee, IOPC). t-NP has emerged as a critical area of research particularly during the COVID-19 pandemic, when the need for remote cognitive assessment and intervention became evident, as a result of social distancing measures.

Recent reviews and meta-analyses have provided support for the diagnostic applicability of t-NP when compared to face-to-face modality (Marra et al., 2020; Alva et al., 2025; see also Sperling et al., 2024), demonstrating the feasibility of t-NP as a viable form of healthcare delivery and offering an alternative to traditional in-person evaluations. However, some limitations including the study publication bias, as well as technical and digital literacy issues were outlined, and additional research on administration modifications to standardize practice is encouraged.

Furthermore, telephone-based cognitive screening (e.g., Montemurro et al., 2023) and web-based computerized testing platforms have been considered as a remote alternative to administering conventional “paper and pencil” tests (e.g., Tsiaras et al., 2024, see Bonvino et al., 2025, for a systematic review and meta-analysis). These tools are not intended as a stand-alone or adjunctive diagnostic device, but can represent a screening phase to identify persons who require a more in-depth clinical assessment (e.g., comprehensive neuropsychological evaluation).

This Research Topic aimed to foster research about the applications of telehealth in the field of neuropsychological assessment and cognitive intervention. It welcomed studies that evaluated the reliability of tele-administered neuropsychological tests compared to the face-to-face counterparts, collected normative data for tele- and web-administered versions of neuropsychological tools, and proposed remote rehabilitation protocols. It covered the areas of both assessment and intervention, as well as different technologies and types of digital support.

In the context of cognitive assessment, three papers in the current issue describe studies that provide new reliability and/or validity, or normative data for remote self-administered digital screening in adults. This type of screening increases the number of people who can be easily reached, including elderly populations, individuals with mobility limitations, or those living in remote and/or geographically disadvantaged areas. In particular, the contribution of Giaquinto et al. provided normative data in the Italian population for a brief computer-based global cognitive assessment, the Self-Administered Tasks Uncovering Risk of Neurodegeneration (SATURN). The open-source test investigates several cognitive domains such as memory, attention, temporal orientation, visuo-constructional abilities, calculation, executive functions, and reading speed. The results support the tool's suitability for self- and remote administration and convergent validity on normative samples. In a similar vein, Huynh et al. highlighted the potential of remote digital cognitive testing as an efficient and cost-effective solution for cognitive screening of people who, for socio-economic or situational reasons, are struggling to access primary care services. The group reported initial evidence on the feasibility and reliability of the BrainCheck, a platform for cognitive assessment in English, that can be remotely self-administered using different devices (smartphone, tablet, laptop). Although remote and self-administered testing may introduce greater variability due to uncontrolled factors in the testing environment, moderate to good agreement between self- and research coordinator-administered versions was found, supporting the feasibility of the tool for remote screening in the healthy population. Finally, Livoti et al. demonstrated the feasibility of web-based testing of age-related changes in multitasking abilities. The self-administered battery consists of three dual-tasks that allow for the assessment of age-related trajectories in dual-task costs, particularly relevant in everyday life contexts across the adult lifespan, as well as the early detection of cognitive impairments.

The results from these studies provide evidence to support the integration of self-administered remote screening tools into clinical workflows to optimize cognitive health outcomes.

Alongside the typical neuropsychological screening and assessment approaches, the contribution by Henneghan et al. expanded the ways in which digital tools can be applied by using a cognitive ecological momentary assessment (EMA) methodology. EMA protocols include daily or almost daily evaluation of subjective and objective cognitive functioning in the natural environment, thus enabling frequent monitoring to track cognitive changes over time, integrating a larger amount of data and improving cognitive impairment detection and treatment, as well as reducing costs. The study reported evidence on the feasibility, test-retest reliability, and convergent validity with baseline clinical cognitive variables for NeuroUX, a cognitive EMA platform for assessing cancer-related cognitive impairment in breast cancer survivors.

Cognitive stimulation and training constitute another promising area for the application of t-NPs. Some evidence comes from the pilot study by Cintoli et al. who compared the feasibility of a remotely administered cognitive stimulation protocol to in-person administration, in individuals with dementia. The results highlighted high levels of satisfaction among both patients and caregivers with the eight weekly 1-h session program.

Finally, a pilot randomized cross-over design study was conducted by Tagliente et al. in persons with Parkinson's disease and mild cognitive impairment to assess the effectiveness of the Neurotablet^®^ platform, a home-based computerized cognitive training program. Significant improvements were found after the experimental training compared to standard care in specific cognitive functions, supporting the potential role of digital, remote interventions in mitigating cognitive decline.

This Research Topic brings together various lines of evidence on the applicability of t-NP within diagnostic and intervention pathways, particularly for individuals who are difficult to reach due to logistical barriers, as well as its potential use as a general screening tool integrated into routine clinical practice.
